# A response to Portman et al. (2025): USGS Bee Lab collaborative science and big data are essential for understanding bee biodiversity

**DOI:** 10.1093/aesa/saaf041

**Published:** 2026-01-09

**Authors:** Neil S Cobb, Brianne Du Clos, Melanie Kammerer, Codey L Mathis, Claus Rasmussen, Laura Russo, Jennifer Schlauch, Erika M Tucker, Tracy Zarrillo

**Affiliations:** Biodiversity Outreach Network, Flagstaff, AZ, USA; Louisiana Universities Marine Consortium, Chauvin, LA, USA; EcoData Technology, Bellefonte, PA, USA; Department of Entomology, Pennsylvania State University, University Park, PA, USA; Department of Agroecology, Aarhus University, Aarhus, Denmark; Department of Ecology and Evolutionary Biology, University of Tennessee, Knoxville, TN, USA; Department of Ecology & Evolutionary Biology, University of California, Irvine, CA, USA; Milwaukee Public Museum, Milwaukee, WI, USA; Department of Entomology, Connecticut Agricultural Experiment Station, New Haven, CT, USA

This article serves as a response to the publication: Zachary M. Portman, Bethanne Bruninga-Socolar, Marissa H. Chase, Tina Harrison, Michael Arduser, Vincent J. Tepedino, Daniel P. Cariveau, Big data, changing taxonomy, and ghost records: permanent preservation of collected specimens is essential for insect monitoring, *Annals of the Entomological Society of America*, Volume 118, Issue 4, July 2025, Pages 331–345, https://doi.org/10.1093/aesa/saaf023.

Portman et al. (2025) address several important topics relevant to an understanding of native bee biodiversity. However, they misuse case studies and mischaracterize the USGS Bee Lab and an occurrence dataset it provides in ways that may lead to misinterpretation of an important USGS program. Here, we address misconceptions from Portman et al. (2025). We correct the factual inaccuracies in Portman et al. (2025) and reiterate the integrity of the USGS Bee Lab at the Eastern Ecological Science Center’s taxonomic work, public data provided through the Global Biodiversity Information Facility (GBIF) and reaffirm its substantial role in supporting biodiversity, inventory, and monitoring efforts. We then underscore the urgent need to promote collaboration in both research and status assessments of wild bees.

## Factual Inaccuracies and Misleading Implications

### USGS Bee Lab Does Not Conduct Monitoring

Portman et al. (2025) misname the USGS Bee Lab at the Eastern Ecological Science Center (aka, “the Bee Lab”) and use its old name, the Bee Inventory & Monitoring Lab (BIML). In 2022, the Bee Lab changed its name to be more reflective of the variety of services it provides. The Bee Lab is a support facility that provides comprehensive resources for native bee inventories, monitoring efforts, and diverse studies and projects through research (eg inventories), education (eg identification workshops), and outreach (eg Discover Life). The Lab has never been mandated to conduct monitoring and does not present itself as a monitoring program.

### Occurrence Data is Not Monitoring Data

Portman et al. examine 3 datasets: the Bee Lab dataset maintained on GBIF titled “Insect Species Occurrence Data from Multiple Projects Worldwide with Focus on Bees and Wasps in North America” (Droege and Maffei 2025) and 2 subsets of previous versions of that dataset (Collado et al. 2019, Kammerer et al. 2020). The Bee Lab dataset comprises records from collaborations with researchers and organizations across North America. The Lab supports a varying number of projects each year with a fluctuating geographic distribution of specimens collected. Although these studies are connected through collaboration with the Bee Lab, the dataset itself comprises discrete, disconnected project data from various individuals and organizations. This published dataset includes lists of species surveys, personal collections, university research records, and more, and employs a broad suite of techniques without standardized or systematic methodology. Portman et al. refer to these data as “monitoring data”; however, the data are unambiguously non-standardized, presence-only occurrence records, and should not be interpreted as monitoring records where there is any expectation of tracking changes in the relative abundance of species across years, without carefully screening appropriate subsets that can be used to track relative abundance of species. This includes *Ceratina strenua* and *Lasioglossum cressonii*, the 2 species they designate as controls; differences in records per year is due to fluctuating sampling effort, and does not reflect relative abundance as they claim. Moreover, this GBIF dataset is continually maintained, with version 1.8 onward explicitly stating that the data provided are occurrence data, and the authors encourage corrections and suggestions for future updates.

“Monitoring” requires repeated surveys over time of the same areas, using the same methods, to minimize variance and bias of signals for tracking changes and temporal trends (Spellerberg 2005, Levenson et al. 2025). From a statistical perspective, the Bee Lab dataset represents opportunistic sampling and is accurately characterized as occurrence data, despite Portman et al. referring to them as monitoring data. The number of species occurrences in the Portman et al. histograms vary widely over the study period. This is expected given the variation in collections and Bee Lab collaborations each year. For example, the difference in *Ceratina floridana* specimens in the GBIF data between 2002 and 2009 (48 individuals), vs. 2010 and 2022 (946 individuals) is due to different studies conducted within the *C. floridana* southeastern range during that timeframe. Analyses of opportunistic occurrence datasets require clear research questions and careful data filtering, but can produce meaningful insights, despite the non-standardized approach to data collection (Collado et al. 2019, Kammerer et al. 2020). In our view referring to the Bee Lab data as monitoring data is inaccurate and supports flawed critiques, which discourage future, proper use of these data. Subsets of the Bee Lab data can be used to track species only after significant filtering to account for standardization of localities, sampling techniques and sampling effort. Collado et al. (2019) and Kammerer et al. (2020) provide excellent examples of how the Bee Lab data should be processed to address specific ecological questions and hypotheses.

### Changing Taxonomic Concepts Can Be Addressed

Portman et al. identified species in 2 genera (*Ceratina* and *Lasioglossum*) that have recently undergone taxonomic changes and may be problematic. Regarding possible mis-identifications of *Lasioglossum* species, Portman et al. did not provide evidence that the Bee Lab misidentified any specimens. Portman et al. state that “without preserved specimens, most of these invalidated records [the taxonomic names of the records they examine] can never be fixed.” and “in most cases, the only way to update species identifications to reflect current taxonomy is to re-examine specimens.” They did not report statistical analyses to support changing taxonomic concepts altering the broader sensitivity or overall conclusion of studies using these data. They did not provide other possible solutions to resolving the distributions of species that have been revised. While specimen records should be provided with the highest quality taxonomic identification at the time of examination, the need to accommodate taxonomic changes has always been a challenge, and is not restricted to bees. Researchers have several options for adjusting existing records to be consistent with accepted taxonomic concepts and/or resolve the true distributions of newly delineated taxa. Problematic or taxonomically unstable species could be excluded from analyses where species identity is of special concern. The focal taxa of Portman et al. were excluded from a recent extinction risk analysis for this reason (Cornelisse et al. 2025). Other solutions include removing all unconfirmed species-level records, pooling problem species to a higher taxonomic unit (eg species cluster or genus), or beginning species-level analyses after a specific nomenclatural revision. Additional options for resolving the known distribution of newly recognized taxa include obtaining specimens from one or more of >350 insect collections in the United States (Cobb et al. 2019) or re-sampling locations of interest for the taxa in question. Science is an inherently iterative process and areas of possible sympatry among new taxonomic delimitations should receive new sampling while prioritizing areas previous research identified as problematic.

Emerging work on data standards and data quality workflows for wild bee occurrence data addresses many of the issues Portman et al. raise about basing conservation decisions on analyses of occurrence data (Dorey et al. 2023, Du Clos et al. 2025). The Bee Lab helped develop “The Wild Bee Data Standard” (WBDS), an adaptation of the Darwin Core standard used by GBIF specifically for wild bee occurrence data (Du Clos et al. 2025). The WBDS provides terms to describe event, occurrence, and taxon information associated with a record; it is flexible and therefore may be appropriately applied by data providers to describe different data collection conditions. The terms include *dwc:verbatimIdentification* and *dwc:identificationQualifier*, which the Lab properly uses to express uncertainty associated with species determinations (Droege and Maffei 2025). When analyzing the USGS Bee Lab data (Droege and Maffei 2025), Portman et al. list 5,601 records of *Ceratina* sp. where they relied on *dwc:verbatimIdentification* to obtain species-level identifications, calling them “morphospecies” (Table 1; Portman et al. 2025). This is 23.6% of the *Ceratina* records analyzed. The definition of *dwc:verbatimIdentification* states that entries provided in this term should not replace the scientific name provided; therefore, assessing species-level identifications based on *dwc:verbatimIdentification* where the scientific name is only provided to the genus level is inappropriate. Additionally, the authors list 3,933 records of *Lasioglossum* sp. in Table 3, where they repeat this approach. This is 18.9% of the “often misidentified” *Lasioglossum* records analyzed. These records should not be used to make claims about species-level or “morphospecies” identifications in Droege and Maffei (2025).

### Sample Retention and Reference Collections

Portman et al. claimed that destruction of samples and lack of a large synoptic collection renders the data “invalidated.” There is no expectation of vouchering in perpetuity when a *dwc:basisOfRecord* is a “preserved specimen”; the term is appropriately applied when the original record was based on the examination of a collected specimen. The reality of working in natural history collections housed in museums or government, academic, and private institutions means making difficult specimen accessioning and deaccessioning choices and making the most of limited resources (Cobb et al. 2019). Portman et al. refer to Packer et al. (2018) specimen retention recommendations; however, Packer et al. (2018) only recommend vouchering focal species exemplars, as is common practice globally. Ergo, one exemplary specimen may represent hundreds as retaining all samples in perpetuity is rarely feasible.

### USGS Bee Lab Taxonomic Expertise and Services

It is critical to provide the highest resolution taxonomic identifications at the time of specimen examination. Sam Droege is a reputable taxonomist for the bees of the eastern US region. Besides identifying most of the >500,000 specimens processed through the Bee Lab, he has identified >19,000 specimens deposited in 34 US natural history collections (Shorthouse 2020). The Lab employs a standard protocol to process species identifiable with existing keys and collaborates directly with specialists on the identification of more problematic species, and where feasible participates in taxonomic revisions (eg *Nomada*). The role of the Bee Lab is to support surveying and research programs, consequently returning most specimens to the original collectors, while also dispersing specimens for long-term reference and/or DNA extractions to >30 natural history collections and research institutes, with >7,000 representative specimens deposited at the Smithsonian (Droege pers. comm.). The Lab has always retained a synoptic reference collection, but never had the funding, space, or staff necessary to maintain a larger collection. In 2024, the Lab added capacity to house ∼90,000 total specimens (Droege pers. comm.).

The merits of the Bee Lab cannot be overstated. They have provided hundreds of thousands of free, reliable bee identifications to >150 stakeholders (including researchers, conservationists, and land managers) from all 50 states. An easy metric to assess the reliability of identifications would be to sample the >19,000 specimens at the 34+ private, state, and federal institutions across the USA that curate specimens donated by the Lab (Shorthouse 2020). The USGS Bee Lab creates, coordinates, and maintains numerous free identification resources, including building and updating the image-rich Discover Life interactive identification keys, and produces thousands of freely available, Creative Commons licensed, high-definition, specimen and character images for public use. In 2021, the Lab started the live and recorded “Learn to Identify Bees” teaching sessions and has shared more than 150 sessions online for easy reference or for anyone who could not attend. The sessions broadly improve taxonomic knowledge in the community and regularly invite expert taxonomists for notoriously difficult bee taxa. Jason Gibbs and Joel Gardner, cited by Portman et al., shared tips and answered questions about the genus *Lasioglossum* over many sessions.

## Philosophical Concerns

One of our most serious concerns is that large datasets may be misrepresented and thus diminish or discourage data sharing, collaborative work, and use of open data sources by researchers in a field that necessitates big data to solve pressing issues. To obtain any real understanding for tracking species distributions, much less population trajectories, we need to collect immense amounts of data to account for existing geographic and temporal biases and filter the data appropriately (Collado et al. 2019, Kammerer et al. 2020). Collaborative science and big data allow for many different groups of scientists to work together to address large-scale population dynamics over space and time; consequently, large data sharing projects should be encouraged, not discouraged.

As a field, we must work together to find solutions to these big problems, and it is important not to misrepresent each other’s work. All efforts to study, inventory, monitor, and conserve wild bees can coexist to help move the field forward. We should be looking for new strategies and technologies to help address the pollinator crisis. Resources provided by qualified scientists greatly benefit the progression of science and conservation. We hope this response helps clarify any misunderstandings that might result from the Portman et al. publication, and to encourage others to share and use openly available data. We suggest Portman et al. publish a review of the specific ways taxonomists and natural history collections are contributing to biodiversity knowledge, thus providing specific justification for more resources as called for in their recommendations. We further encourage them to incorporate all the ways the Bee Lab has contributed to taxonomy and supported natural history collections.

## Author contributions

Neil S. Cobb (Conceptualization [equal], Supervision, Writing—original draft [equal], Writing—review and editing [equal]), Brianne Du Clos (Conceptualization [equal], Writing—original draft [equal], Writing—review and editing [equal]), Melanie Kam­merer (Conceptualization [equal], Writing—original draft [equal], Writing—review and editing [equal]), Codey Mathis (Conceptualization [equal], Writing—original draft [equal], Writing—review and editing [equal]), Claus Rasmussen (Con­ceptualization [equal], Writing—original draft [equal], Writ­ing—review and editing [equal]), Laura Russo (Conceptualization [equal], Writing—original draft [equal], Writing—review and editing [equal]), Jennifer Schlauch (Conceptualization [equal], Writing—original draft [equal], Writing—review and editing [equal]), Erika M. Tucker (Conceptualization [equal], Writ­ing—original draft [equal], Writing—review and editing [equal]), and Tracy Zarrillo (Conceptualization [equal], Writing—orig­inal draft [equal], Writing—review and editing [equal])
